# Associations of periconception dietary glycemic index and load with fertility in women and men: a study among couples in the general population

**DOI:** 10.1186/s12916-024-03718-z

**Published:** 2024-10-29

**Authors:** Mireille C. Schipper, Aline J. Boxem, Sophia M. Blaauwendraad, Annemarie G. M. G. J. Mulders, Vincent W. V. Jaddoe, Romy Gaillard

**Affiliations:** 1 The Generation R Study Group, Erasmus MC, University Medical Center, PO Box 2040, Rotterdam, CA 3000 the Netherlands; 2 Department of Pediatrics, Sophia’s Children’s Hospital, Erasmus MC, University Medical Center, Rotterdam, the Netherlands; 3 Department of Obstetrics and Gynaecology, Erasmus MC, University Medical Center, Rotterdam, the Netherlands

**Keywords:** Glycemic index, Glycemic load, Fertility, Periconception period, Carbohydrates

## Abstract

**Background:**

The dietary glycemic index (GI) and load (GL) reflect carbohydrate quality and quantity, potentially impacting fertility through modulation of insulin sensitivity and generation of oxidative stress. While fertility is influenced by both women and men, reproductive research often emphasizes maternal factors. We first examined periconception dietary intake in both women and male partners, and subsequent associations of dietary GI and GL with fecundability and subfertility.

**Methods:**

Among 830 women and 651 male partners, participating in a population-based prospective cohort study from preconception onwards, we assessed periconception dietary intake and calculated GI and GL, using a food frequency questionnaire (FFQ) at median 12.4 weeks gestation (95% range 10.9, 18.4). Information on time to pregnancy was obtained through questionnaires, with subfertility defined as a time to pregnancy ≥ 12 months or use of assisted reproductive technology.

**Results:**

In the periconception period, mean energy intake in women was 1870 kcal (SD: 500; 46% carbohydrates, 16% protein, 33% fat; dietary GI 56.2 (SD: 3.5) and GL 141.4 (SD: 67.4)). Mean energy intake in men was 2350 kcal (SD: 591; 43% carbohydrates, 16% protein, 33% fat; dietary GI 56.8 (SD: 3.2) and GL 156.7 (SD: 75.4)). Median time to pregnancy was 4.8 months (IQR: 1.2, 16.4), with 30.6% of 830 women experiencing subfertility. Dietary GI and GL were not associated with fertility outcomes in women. In men, higher dietary GI and GL across the full range were associated with decreased fecundability, after adjusting for socio-demographic and lifestyle factors, as well as dietary GI or GL of female partners [FR: 0.91, 95% CI 0.83, 0.99; FR: 0.90, 95% CI 0.81, 0.99, per SDS increase in dietary GI and GL, respectively]. When assessing the combined influence of dietary GI clinical categories in women and men, both partners adhering to a low GI diet tended to be associated with increased fecundability, but not with subfertility risk.

**Conclusions:**

Suboptimal periconception carbohydrate intake may be negatively associated with male fertility, but not with fertility outcomes in women. Further studies are needed to assess whether a lower GI and GL diet is a feasible lifestyle intervention to improve couples fertility.

**Supplementary Information:**

The online version contains supplementary material available at 10.1186/s12916-024-03718-z.

## Background

Subfertility, defined as the inability to conceive after 12 months of regular, unprotected intercourse, is a prevalent and growing concern globally and affects millions of couples worldwide [1–3]. Most influential factors related to subfertility, such as age and genetic predisposition, are non-modifiable. Because fertility treatments are expensive, have modest success rates, and create a physiological burden for those affected, it is important to identify modifiable factors related to subfertility [4].

In recent decades, the importance of diet as a potential modifiable risk factor in reproductive health has been increasingly acknowledged [5]. Elevated glucose concentrations, directly linked to carbohydrate intake and carbohydrate quality, have gained particular attention when assessing the impact of diet on reproductive health. High glucose levels can negatively influence fertility through modulation of insulin sensitivity, leading to hormonal disruptions and impaired ovarian function [6]. Additionally, hyperglycemia is associated with oxidative stress, characterized by the production of reactive oxygen species (ROS), which play a key role in the pathogenesis of subfertility [7]. Two dietary measures used to assess and quantify the carbohydrate intake and quality, as well as the postprandial glycemic response to carbohydrate intake, are the glycemic index (GI) and glycemic load (GL) [8]. A low GI diet can be achieved by consuming carbohydrate-containing foods that are less likely to increase blood sugar levels, referred to as low GI products, while avoiding those with a high GI. For a low GL diet, daily quantity of carbohydrates is additionally taken into account. A meta-analysis of eight studies focusing on women with overweight and obesity has demonstrated that adhering to a low GI diet may substantially mitigate risk of polycystic ovary syndrome (PCOS), improving both clinical and biochemical features [9]. In a small-scale randomized controlled study with overweight or obese subfertile women undergoing in vitro fertilization (IVF), a hypocaloric low GI diet demonstrated improved oocyte development and pregnancy rate [10]. Yet, majority of studies investigating the impact of a low GI diet are performed in at-risk populations, with limited research conducted in the general population. A prospective analysis of 18,555 healthy women revealed a positive association between total carbohydrate intake, dietary GL, and GI with infertility, the latter evident solely among nulliparous women [11]. Two web-based preconception cohorts in Denmark and North America found that high GL and added sugar diets in women were associated with a slight reduction in fecundability, reflecting the cycle-specific probability of conception [12, 13]. Despite the potential impact of male health and behavior on reproductive outcomes, these factors are often overlooked in reproductive health research. To date, no studies have explored the association between dietary GI or GL and fecundability in men. In highly selected and at-risk populations, research on male reproductive health suggests that dietary GI and GL may influence sperm quality and function. A sub-study conducted within the FERTINUTS trial among 106 healthy non-smoking men following a Western-style diet found inconsistent effects of dietary GI on sperm quality meters, while dietary GL was positively associated with sperm quality parameters [14]. Contrary, a cross-sectional study among 322 non-smoking men attending an IVF clinic showed that a diet with a higher GI was beneficial for sperm quality parameters, whereas a higher dietary GL was associated with reduced sperm quality parameters [15]. Yet, the population of men attending an IVF clinic is markedly different from the general population, as these men often have underlying reproductive issues or are undergoing treatment, which may affect both their diet as well as their sperm quality. This limits the generalizability of findings from IVF populations to the general population. Exploring the independent and combined effects of dietary GI and GL in both women and men on their fertility and time to pregnancy within the general population is crucial for understanding reproductive health complexities and designing comprehensive preconception care strategies.

Therefore, in a population-based prospective cohort study from the preconception period onwards, we first assessed dietary intake of women and male partners during the periconception period, and second, explored associations of periconception dietary GI and GL with fecundability and subfertility.

## Methods

### Study population

This study was embedded in the Generation R *Next* Study, a population-based prospective cohort study from the preconception period onwards in the city of Rotterdam, the Netherlands, and is part of the Generation R Study Programme [16]. The general aim of this study is to identify preconception and early-pregnancy determinants of fertility, embryonic development, and childhood outcomes. Women and their partners from the general population were eligible if they were ≥ 18 years old, residing in Rotterdam, and actively trying to conceive or already pregnant. Couples were included in the preconception period or during pregnancy between 2017 and 2021. While enrollment aimed for inclusion during preconception or early pregnancy, enrollment was allowed until delivery. In total, 33.2% and 52.8% of all inclusions were during the preconception period or in the first trimester of pregnancy, respectively. Study approval was obtained by the Medical Ethical Committee of the Erasmus University Medical Centre, Rotterdam (MEC 2016–589, NL57828.078.16). Written informed consent was obtained from participating women and men. In total, there were 4036 participant episodes from 3604 unique women, leading to 3577 pregnancies of 3200 unique women at the end of the study. Dietary intake data was collected using a food frequency questionnaire (FFQ) distributed to a smaller number of couples during early pregnancy, resulting in the current study being conducted within a subgroup of the original population. In total, 1054 unique women that enrolled between 2017 and 2021 provided information on dietary intake. Of these, 830 unique women provided information on time to pregnancy, with 651 women having male partners who also provided information on dietary intake. Details are present in the flowchart (Additional file 1: Fig. S1).

### Periconception dietary glycemic index and load

We obtained information on periconception dietary intake of women and men during early pregnancy at a median of 12.4 weeks of gestation (95% range 10.9, 18.4), using the Dutch version of the semi-quantitative HELIUS (HEalthy LIfe in an Urban Setting) FFQ [17]. This FFQ was developed at the Amsterdam UMC in collaboration with the National Institute for Public Health and the Environment (RIVM) and Wageningen University [17]. Women and men were asked to report the frequency of consumption of > 200 food items over the past 4 weeks. Ten possibilities of frequency were offered, ranging from never to 7 times per week. Following questions on consumption frequency, women and men indicated the typical quantity consumed on a consumption day, utilizing standardized units, household measures, or visual aids such as colored photographs depicting various portion sizes. Were possible, food items were grouped according to the typical order of daily consumption; the questionnaire begins with foods typically eaten at breakfast. For conversion into nutrients, each food item in the questionnaire was linked with one or more foods from the Dutch food composition Table 2011 (Netherlands Nutrition Centre (2011) NEVO: Dutch food composition database 2011. Netherlands Nutrition Center, the Hague). For food items consisting of more than a single food, nutrient values were calculated by weighing the individual foods according to their frequency of consumption (as percentage of consumption days). Average daily energy and nutrient intakes were calculated by multiplying the frequency of consumption by the consumed amounts and nutrient content per item. To correct for potential under- or over-reporting, we excluded participants with implausible habitual dietary intake consisting of an energy intake < 800 and > 4000 kcal for men and < 500 and > 3500 kcal for women (2.7% and 3.3% for women and men, respectively) [18]. Additional information on the processing of the dietary intake data is described in Additional file 1: Supplementary Information S1. We used this dietary assessment as a measurement of periconception diet, as previous literature studies have shown that maternal dietary patterns tend to remain stable during the transition from preconception to the first weeks of pregnancy [19, 20]. Within a large cohort of 12,583 UK women, minimal dietary pattern changes were observed from preconception to pregnancy, as determined by principal component analyses [19]. Similarly, a longitudinal study among healthy Spanish females found no significant change in dietary patterns from preconception to pregnancy, persisting 6 months postpartum [20].

Next, we calculated periconception dietary GI and GL of women and men. We considered dietary GI as our main exposure, as dietary GI provides information on the quality of the glycemic response to a carbohydrate-containing food product and is more often used in intervention studies and clinical settings [21, 22]. We included dietary GL as a secondary exposure, as this measure takes the amount of carbohydrate into account and provides additional information on postprandial glucose concentrations, but this measure may be more prone to measurement error [8, 23, 24]. To calculate periconception dietary GI and GL, GI values were assigned to each individual food item in the FFQ. GI values were obtained from the GI database on the Dutch diet published by the Medical Research Council Human Nutrition Research (MRC HNR), Cambridge, UK, using glucose as a reference (GI for glucose equal to 100) [25]. This database was developed using a standardized approach of calculating dietary GI and GL to facilitate research into the health effect of dietary GI and GL [25]. Using this database, we obtained matches for 75.2% of the food items. For the food items that could not directly be matched in the database, GI values for similar food items were obtained from proxies (72.9%) or from GI databases of the MRC HNR of other countries (11.9%). If no equivalent food item was available for a food item, an arbitrary value of 70 was assigned according to the procedure developed by the MRC HNR (15.3%) [25, 26]. Food items without any carbohydrates were excluded from the calculations. An overview of matching food items of the FFQ with GI values can be found in Additional file 1: Supplementary Information S1. The mean dietary GI per day was calculated by summing the product of the carbohydrate intake of each food item with its GI value, which was divided by the total amount of carbohydrates consumed per day. The mean dietary GL was calculated by summing the product of the carbohydrate intake of each food item with the GI value of that specific food item [8, 23, 27]. We categorized periconception dietary GI and GL of both women and men into quartiles and calculated standard deviation scores (SDS). This approach allowed us to analyze the associations in a dose-dependent manner, examining potential trends across the full range of dietary GI and GL. Intervention studies stimulate a low GI diet by recommending an exchange of high GI products for low GI products, which results in a low mean dietary GI [21, 28]. In line with these studies, we aimed to explore the effects of a low GI diet on fecundability and subfertility as a secondary analysis. We categorized the mean dietary GI per day into a low, normal, and high GI diet, using similar cut-offs as used for individual food products [low GI diet (≤ 55), a normal GI diet (56–69), and a high GI diet (≥ 70)] [21, 27]. In multivariate models, all models were adjusted for total energy intake (kcal).

### Time to pregnancy

Time to pregnancy and mode of conception were assessed through questionnaires in the preconception period and early pregnancy. We used questions regarding the date at which women started trying to conceive and refrained from using contraceptives (start date actively pursuing pregnancy) and the date of assisted reproductive technology, including intrauterine insemination (IUI), ovulation induction, in vitro fertilization (IVF), and intracytoplasmic sperm injection (ICSI). The first day of the last menstrual period was obtained from the obstetric caregiver. Time to pregnancy was calculated from the start date of actively pursuing pregnancy and their first day of last menstrual period. In women who used assisted reproductive technologies, we added 12 months to their time to pregnancy, as fertility treatments usually start after 1 year of not conceiving [29]. Fecundability was defined as the probability of conceiving within 1 month. Time to pregnancy was categorized in two groups: < 12 months (fertile) and ≥ 12 months (subfertile). Women who underwent assisted reproductive technology were included in the subfertile group.

### Covariates

Information on age, ethnicity, and highest education level was obtained through questionnaires at enrollment. Ethnicity was assessed by the country of birth of the participant and her/his parents. If one of the participants’ parents was born abroad, participants were classified as of non-Dutch ethnic origin [30, 31]. Information on smoking, alcohol use, drug use, folic acid supplement use, parity, pre-existent diabetes mellitus or gestational diabetes, and vomiting or nausea during the first trimester of pregnancy were assessed through questionnaires filled in during the preconception period and/or in early pregnancy. Preconception body mass index (BMI) in women and BMI in men were assessed either via clinical measurements of height and weight at time of enrollment and/or first trimester visits without shoes and heavy clothing, or obtained through questionnaires. BMI was categorized into underweight/normal weight (≤ 24.9 kg/m^2^) and overweight/obesity (≥ 25 kg/m^2^).

### Statistical analysis

First, we performed a non-response analysis comparing characteristics of women and men with and without information available on dietary intake using* t*-tests, Mann–Whitney *U* tests, chi-square tests, or Fisher’s exact tests. Second, to gain insight into the adequacy of the dietary intakes of women and men during the periconception period, nutrient intake data were compared to the Dutch dietary reference values established by the Dutch Health Council for adults [32–35]. An overview of the dietary reference values can be found in Additional file 1: Supplementary Information S1. The percentage of women and men with mean dietary intake below the estimated average requirement (EAR), or when no EAR value was available, below the adequate intake (AI), was calculated and reported. We further compared population characteristics according to women and men’s dietary GI quartiles, using one-way ANOVA tests, Kruskal–Wallis tests, chi-square tests, or Fisher’s exact tests.

Third, we examined associations of GI and GL in SDS and quartiles with fecundability in women and men using Cox proportional hazard models (R package *survival*). The survival outcome was conception. The time variable was time to pregnancy (months/28 days). We checked the proportional hazard assumptions of the covariates using Schoenfeld residuals, assessed linearity of all associations using Martingale residuals, and assessed influentials using deviance residuals. Violations were detected for pre-pregnancy BMI and parity in women. To address this, time-transformed covariates were included to the models. The resulting hazard ratios from the Cox proportional hazard models represent the fecundability ratios. We examined the associations of GI and GL in SDS and quartiles with the odds of subfertility in women and men using logistic regression models. We specifically aimed to identify independent and combined effects of GI and GL in women and men on fertility outcomes, and therefore examined associations of GI and GL in SDS with fertility outcomes in women and men singularly and simultaneously. The statistical interaction terms between dietary GI and GL of women and men were not statistically significant. All regression models were first analyzed in univariate models and second in multivariate models with adjustment for potential confounders. Potential confounders were selected a priori based on a directed acyclic graph (DAG) and associations of the exposure and outcome in existing literature (Additional file 1: Fig. S2) [5, 36–39]. We adjusted the models for women for age, ethnicity, educational level, alcohol use, smoking, preconception BMI, parity, and total energy intake. The models for men were adjusted for age, ethnicity, educational level, alcohol use, smoking, BMI, and total energy intake. Combined models for both women and men included all above mentioned covariates. We explored inclusion of more dietary factors; however, due to multicollinearity issues, these were not included in the final models.

Fourth, to enable clinical translation, we explored effects of adherence to a low GI diet as compared to a normal GI diet in women and men, on time to pregnancy using Cox proportional hazard models, and subfertility using logistic regression models. To assess the combined impact of both partners adhering to a low GI diet, we created a variable representing GI clinical categories based on the levels of both partners [both partners normal GI diet, women low GI diet and men normal GI diet, women normal GI diet and men low GI diet, both partners low GI diet].

We performed three sensitivity analyses. First, we repeated analyses restricted to women and men with a (pre-pregnancy) BMI ≥ 25 kg/m^2^, as they represent a population at higher risk of impaired glucose metabolism who may be more prone to adverse effects of a higher dietary GI and GL diet. Second, we repeated analyses excluding women and men with pre-existent diabetes mellitus or gestational diabetes (in women), as these conditions are associated with alterations in glucose metabolism, insulin resistance, and hormonal imbalances, all of which can impact fertility. Third, we repeated our analysis in women stratified for parity, as previous literature has reported that parity may mediate the effect of dietary GI and GL on ovulatory infertility [11].

Missing values were imputed using Multiple Imputation by Chained Equations to reduce potential bias due to missing values of covariates (R package *mice*). Pooled results were reported. The percentage of missing values for covariates ranged from 0 to 18.9%. *P* values of < 0.05 were considered significant. Analyses were performed using R Statistical Software version 4.2.1, SPSS version 28.0.1.0, and SAS version 9.4.

## Results

### Population and dietary characteristics

Women had a mean age of 32.1 years (SD: 3.9) and a median pre-pregnancy BMI of 23.0 kg/m^2^ (IQR: 21.1, 25.3). Men had mean age of 34.2 years (SD: 5.0) and a median BMI of 24.6 kg/m^2^ (IQR: 22.7, 26.8). The majority of women and men were of Dutch nationality, highly educated, nulliparous (in women), and nonsmokers. Median time to pregnancy was 4.8 months (IQR: 1.2, 16.4), with 30.6% of 830 women experiencing subfertility (≥ 12 months to conceive) (Table 1).

**Table 1:** Population characteristics

	Women *n* = 830	Men *n* = 651
Population characteristics		
Age at dietary intake assessment, mean (SD), years	32.1 (3.9)	34.2 (5.0)
Gestational age at dietary intake assessment, median, (95% range), weeks	12.4 (10.9, 18.4)	na
Ethnicity, % (*n*)		
Dutch	69.0 (570)	74.8 (485)
European	10.0 (83)	7.9 (51)
Non-European	20.9 (173)	17.3 (112)
Education level, high, % (*n*)	82.5 (675)	75.0 (487)
(Pre-pregnancy) body mass index, median (IQR), kg/m^2^	23.0 (21.1, 25.3)	24.6 (22.7, 26.8)
Overweight/obesity, % (*n*)	27.6 (222)	44.3 (286)
Smoking before pregnancy, % (*n*)	41.7 (315)	48.4 (313)
Alcohol use before pregnancy, % (*n*)	85.1 (701)	92.1 (596)
Drug use before pregnancy, % (*n*)	8.7 (72)	na
Periconception folic acid supplement use, % (*n*)	99.5 (804)	na
Parity, nulliparous, % (*n*)	72.4 (594)	na
Pre-existing or gestational diabetes, % (*n*)	6.8 (56)	0.9 (6)
Daily nausea and vomiting during early pregnancy, % (*n*)	2.5 (20)	na
Previous miscarriage, % (*n*)	19.1 (142)	na
Glycemic index, mean (SD)	56.2 (3.5)	56.8 (3.2)
Glycemic load, mean (SD)	141.4 (67.4)	156.7 (75.4)
Low glycemic index diet, % (*n*)	39.5 (328)	32.0 (208)
Outcome characteristics		
Time to pregnancy, median (IQR), months	4.8 (1.2, 16.4)	na
Time to pregnancy ≥ 12 months (subfertility), % (*n*)	30.6 (254)	na
Pregnancy results of fertility treatment, % (*n*)	13.2 (108)	na

In the periconception period, mean energy intake in women was 1870 kcal (SD: 500; 46% carbohydrates, 16% protein, 33% fat). Mean energy intake in men was 2350 kcal (SD: 591; 43% carbohydrates, 16% protein, 33% fat). A substantial proportion of women and men demonstrated dietary intakes below EAR/AI reference values, particularly in total dietary fiber, calcium, retinol activity equivalents, dietary folate equivalents, magnesium, vitamin D, and omega-3 poly-unsaturated fatty acid (PUFA) intake (Table 2). Mean dietary GI and GL in women were 56.2 (SD: 3.5) and 141.4 (SD: 67.4), and mean dietary GI and GL in men were 56.8 (SD: 3.2) and 156.7 (SD: 75.4), respectively. 39.5% of women and 32.0% of men consumed a low GI diet, while no individuals consumed a high GI diet (Table 1).

**Table 2:** Periconception dietary intake of women and men

	Women *n* = 830	< EAR/AI (%)	Men *n* = 651	< EAR/AI (%)
Macronutrients				
Energy, mean (SD), MJ	7.8 (2.1)		9.9 (2.5)	
Energy, mean (SD), kcal	1870.0 (499.5)		2350.3 (591.4)	
Total carbohydrates, mean (SD), g	215.1 (61.7)		250.4 (68.8)	
Carbohydrates, % of energy intake, mean (SD)	46.2 (5.8)		42.8 (5.7)	
Total mono and di-saccharides, mean (SD), g	95.8 (37.5)		96.6 (37.4)	
Total poly-saccharides, mean (SD), g	119.0 (36.0)		153.5 (45.2)	
Total dietary fiber, mean (SD), g	23.0 (6.9)	74.8	26.1 (8.3)	86.9
Total protein, mean (SD), g	74.5 (22.2)	9.7	95.7 (26.5)	6.2
Protein, % of energy intake, mean (SD)	16.0 (2.6)		16.4 (2.6)	
Total animal protein, mean (SD), g	42.0 (18.2)		55.4 (22.3)	
Total vegetable protein, mean (SD), g	32.5 (10.4)		40.3 (13.3)	
Total fat, mean (SD), g	68.2 (22.5)		88.0 (28.2)	
Fat, % of energy intake, mean (SD)	32.6 (5.0)	0.8	33.4 (4.8)	0.2
SFA, mean (SD), g	26.5 (10.0)		33.1 (11.9)	
MUFA, mean (SD), g	25.0 (8.7)		33.8 (11.4)	
PUFA, mean (SD), g	14.6 (5.8)		18.5 (7.0)	
Trans fat, mean (SD), g	1.0 (0.4)		1.1 (0.4)	
Omega-3 PUFA, mean (SD), g	1.8 (0.8)		2.2 (0.9)	
ALA, mean (SD), g	1.5 (0.7)		1.8 (0.8)	
DHA, mean (SD), g	0.1 (0.1)	60.0	0.1 (0.1)	52.5
EPA, mean (SD), g	0.1 (0.1)		0.1 (0.1)	
Omega-6 PUFA mean (SD), g	12.4 (5.0)		15.9 (6.1)	
Micronutrients				
Calcium, mean (SD), mg	976.3 (410.1)	29.8	1067.8 (434.3)	25.2
Iron, mean (SD), mg	9.8 (2.7)	13.5	12.0 (3.2)	1.8
Retinol activity equivalent, mean (SD), µg	581.9 (267.4)	47.7	787.1 (634.7)	48.7
Vitamin B1, mean (SD), mg	0.9 (0.4)		1.1 (0.3)	
Vitamin B2, mean (SD), mg	1.3 (0.6)		1.5 (0.5)	
Vitamin B6, mean (SD), mg	1.7 (0.6)	11.3	2.0 (0.6)	3.7
Vitamin B12, mean (SD), µg	4.2 (2.8)	12.2	5.7 (3.3)	4.1
Dietary folate equivalents, mean (SD), µg	258.8 (75.5)	21.4	286.7 (78.3)	14.0
Phosphorus, mean (SD), mg	1410.3 (455.1)		1717.8 (499.1)	
Magnesium, mean (SD), mg	325.8 (96.1)	41.4	387.6 (113.5)	38.1
Zinc, mean (SD), mg	9.8 (3.0)	6.6	12.5 (3.6)	2.9
Vitamin C, mean (SD), mg	138.6 (58.9)	3.3	121.2 (50.4)	7.5
Vitamin D, mean (SD), µg	2.6 (1.3)	100	3.4 (1.7)	99.4

*Abbreviations*: *SFA* Saturated fatty acids, *MUFA* Mono-unsaturated fatty acids, *PUFA* Poly-unsaturated fatty acids, *ALA* Alpha-linolenic acid, *DHA* Docosahexaenoic acid, *EPA* Eicosapentaenoic acid, *EAR* Estimated average requirement, *AI* Adequate intake

Tables S1 and S2 show population characteristics stratified by dietary GI. Women within the higher dietary GI quartiles were more likely to be of younger age, of non-European nationality, lower educated, and had a higher pre-pregnancy BMI (Additional file 1: Table S1). Men within the higher dietary GI quartiles were more likely to be lower educated, smokers, and had a higher BMI (Additional file 1: Table S2). Non-response analyses revealed that women and men with dietary intake data available were more likely to be of Dutch nationality, highly educated, nulliparous (in women), nonsmokers, alcohol users, and exhibited a lower (pre-pregnancy) BMI, compared to those without dietary intake data (Additional file 1: Table S3).

### Dietary glycemic index and load, time to pregnancy, and subfertility

Higher dietary GI and GL in women across the full range were not associated with fecundability. In men, higher dietary GI and GL across the full range tended to be associated with decreased fecundability after adjusting for socio-demographic and lifestyle factors [adjusted FR: 0.92, 95% CI 0.85, 1.00, per SDS increase in dietary GI; adjusted FR: 0.92, 95% CI 0.84, 1.02, per SDS increase in dietary GL]. In combined models, higher dietary GI and GL in men across the full range were associated with decreased fecundability after adjusting for socio-demographic and lifestyle factors and dietary GI or GL of female partners [adjusted FR: 0.91, 95% CI 0.83, 0.99, per SDS increase in dietary GI; adjusted FR: 0.90, 95% CI 0.81, 0.99, per SDS increase in dietary GL] (Table 3). No significant associations of dietary GI and GL in quartiles with fecundability were observed in either women or men (Additional file 1: Table S4). Although higher dietary GI and GL across the full range or in quartiles were not significantly associated with an increased risk of subfertility in women and men, higher dietary GL in men across the full range exhibited a trend towards an association with an increased risk of subfertility in combined models (Table 4, Additional file 1: Table S5).

**Table 3:** Fecundability ratios for periconception dietary glycemic index and load

	Separate models				Combined models		
	Fecundability ratio (95% CI)				Fecundability ratio (95% CI)		
		*n*	Unadjusted model	Confounder model		*n*	Confounder model
Glycemic index (SDS)	Women	830	1.01 (0.95, 1.08)	0.97 (0.90, 1.04)	Women	651	0.98 (0.89, 1.07)
	Men	651	0.94 (0.87, 1.02)	0.92 (0.85, 1.00)	Men	651	0.91 (0.83, 0.99)*
Glycemic load (SDS)	Women	830	0.95 (0.89, 1.02)	0.95 (0.87, 1.04)	Women	651	1.02 (0.91, 1.14)
	Men	651	0.98 (0.91, 1.06)	0.92 (0.84, 1.02)	Men	651	0.90 (0.81, 0.99)*

Values represent the fecundability per SDS (standard deviation score) increase in the dietary glycemic index or load for women and men. Fecundability represents the probability of conceiving within 1 month (28 days). Models were analyzed using Cox proportional hazard models. Fecundability ratios were derived from the hazard ratios of the Cox proportional hazard models. Separate models consider the dietary glycemic index or load of women or men independently. Combined models consider the dietary glycemic index or load of both women and men together in one model

Confounder models in women included age, ethnicity, educational level, alcohol use, smoking, pre-pregnancy body mass index, parity, and total energy intake of women

Confounder models in men included age, ethnicity, educational level, alcohol use, smoking, body mass index, and total energy intake of men

Combined confounder models included all confounders listed for both women and men

^*^P value < 0.05

**Table 4:** Associations of periconception dietary glycemic index and load with odds of subfertility

	Separate models				Combined models		
	Odds ratio (95% CI)				Odds ratio (95% CI)		
		*n*	Unadjusted model	Confounder model		*n*	Confounder model
Glycemic index (SDS)	Women	830	0.99 (0.86, 1.15)	1.04 (0.88, 1.22)	Women	651	1.03 (0.83, 1.27)
	Men	651	1.16 (0.98, 1.37)	1.17 (0.97, 1.41)	Men	651	1.17 (0.95, 1.45)
Glycemic load (SDS)	Women	830	1.13 (0.97, 1.30)	1.14 (0.94, 1.37)	Women	651	0.89 (0.69, 1.16)
	Men	651	1.11 (0.94, 1.30)	1.15 (0.94, 1.42)	Men	651	1.26 (0.99, 1.59)

Values represent the odds of subfertility (≥ 12 months to conceive) per SDS (standard deviation score) increase in the dietary glycemic index or load for women and men. Models were analyzed using logistic regression models. Separate models consider the dietary glycemic index or load of women and men independently. Combined models consider the dietary glycemic index or load of women and men together in one model

Confounder models in women included age, ethnicity, educational level, alcohol use, smoking, pre-pregnancy body mass index, parity, and total energy intake of women

Confounder models in men included age, ethnicity, educational level, alcohol use, smoking, body mass index, and total energy intake of men

Combined confounder models included all confounders listed for both women and men

### Low glycemic index diet compared to a normal glycemic index diet

**Fig. 1:**
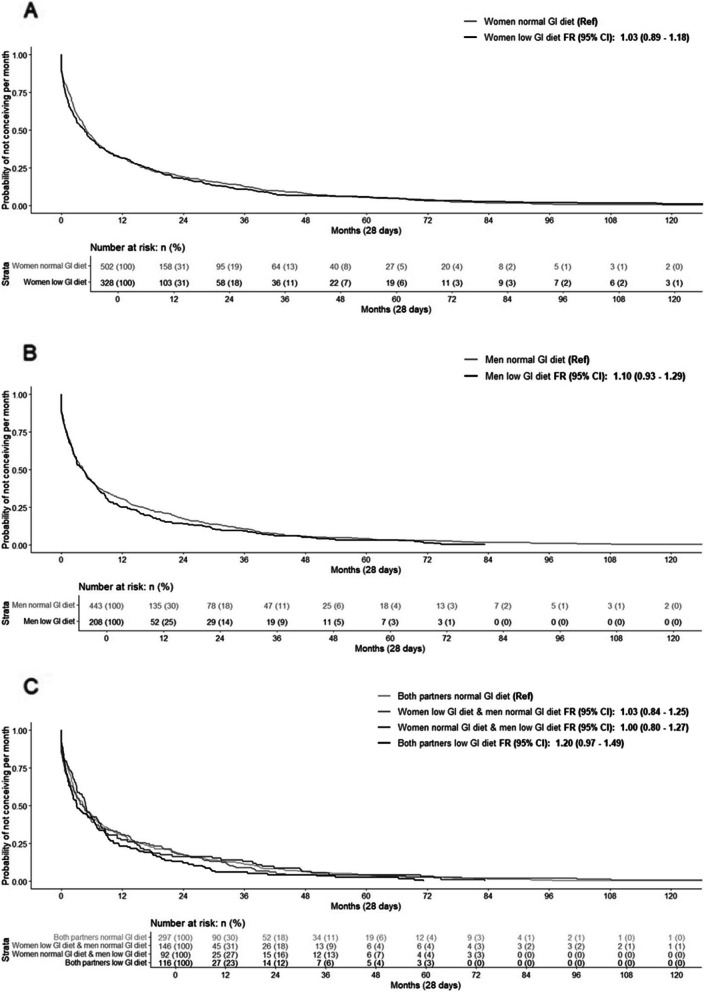
Kaplan–Meier survival curves for dietary glycemic index clinical categories. The survival curves represent the survival probability (probability of not conceiving per month (28 days)) by **A** dietary glycemic index clinical categories in women, **B** dietary glycemic index clinical categories in men, and **C** combined dietary glycemic index clinical categories of both partners. Fecundability ratios (FRs) are shown within the figures and represent the fecundability when compared to the reference category. Fecundability represents the probability of conceiving within 1 month (28 days). Fecundability ratios were derived from the hazard ratios of the Cox proportional hazard models. Survival curves and fecundability ratios are displayed for unadjusted models

No significant effect on fecundability or risk of subfertility was observed when comparing women and men adhering to a low GI diet with those following a normal GI diet. When assessing the combined influence of dietary GI clinical categories in both women and men, both partners adhering to a low GI diet tended to be associated with increased fecundability [unadjusted FR: 1.20, 95% CI 0.97, 1.49; adjusted FR: 1.25, 95% CI 0.99, 1.58, compared to both partners adhering to a normal GI diet], but not with subfertility risk (Fig. 1, Additional file 1: Table S6). These effects were not strongly affected by adjustment for socio-demographic and lifestyle factors (Additional file 1: Table S6).

### Sensitivity analyses

When repeating our analyses for individuals with overweight or obesity, and excluding those with pre-existing or gestational diabetes, we observed largely similar effect estimates (Additional file 1: Tables S7–S8). When stratifying by parity, higher dietary GL in women across the full range was associated with decreased fecundability and elevated risk of subfertility in nulliparous women, solely in unadjusted models [unadjusted FR: 0.92, 95% CI 0.85, 1.00; unadjusted OR: 1.22, 95% CI 1.03, 1.45 per SDS increase in dietary GL]. This effect did not persist after adjusting for socio-demographic and lifestyle factors, although a similar direction of effect was observed (Additional file 1: Table S9).

## Discussion

In this population-based prospective cohort study, we observed a substantial proportion of women and men exhibiting dietary intake values below EAR/AI reference values during the periconception period, suggesting potential nutritional inadequacies. Higher dietary GI and GL in men were associated with reduced fecundability, but not in women. From a clinical perspective, consumption of a low GI diet by both men and women as compared to a normal GI diet tended to be associated with increased fecundability. No associations with subfertility risk were present.

### Main findings

There is increasing recognition for the importance of lifestyle and nutrition in reproductive health. Poor adherence to dietary guidelines or nutritional recommendations during the preconception period and throughout pregnancy can negatively impact fertility, pregnancy, and birth outcomes, as well as the future health of the offspring [40, 41]. This not only holds for macronutrients, but micronutrients as well. A systematic review exploring adequacy of preconception dietary intake found that folate and vitamin E were most frequently reported as being inadequate in diets of preconceptual women, while protein intake was likely to exceed daily recommendations [42]. Among a more recently published cross-sectional study including Canadian women planning to conceive, mean dietary intake was below dietary reference intakes for carbohydrates, vitamins D and E, and above dietary reference intakes for total fat and folic acid [43]. Notably, majority of studies exploring adequacy of preconception diet considered non-European women. Since some countries have specific dietary reference values tailored to their populations, direct comparison may not always be straightforward. We found that a substantial proportion of women and men demonstrated dietary intakes below EAR/AI reference values. More than half of women and men exhibited dietary fiber, omega-3 PUFA, and vitamin D intake below the EAR/AI, suggesting potential nutritional inadequacies within our study population. Women and men consumed 46% and 43% of their total calories from carbohydrates, respectively, which is in line with the recommended carbohydrate intake outlined by Dutch dietary guidelines. The mean dietary GI and GL in our study population were 56.2 and 141.4 for women and 56.8 and 156.7 for men, respectively, with no individuals adhering to a high GI diet.

To date, three prospective cohort studies have examined associations between dietary GI or GL in women and fertility. A study within the Nurses’ Health Study II cohort, involving 18,555 healthy women, identified a positive association between dietary GI and GL and infertility attributed to ovulatory disorders. Notably, the association for dietary GI was observed exclusively among nulliparous women [11]. In two web-based preconception cohorts, “Snart Foraeldre” (SF) from Denmark and “Pregnancy Study Online” (PRESTO) from North America, diets higher in GL were associated with reduced fecundability in women [12]. While not directly evaluating dietary GI or GL, a multi-center pregnancy-based cohort study of 5598 nulliparous women with low-risk singleton pregnancies demonstrated that increased consumption of fast food items, which are commonly linked to high GI and GL values, was associated with a modest increase in time to pregnancy and subfertility [44]. Contrary to prior research, we did not find any association between dietary GI or GL and fecundability or subfertility risk in the full group of women. We observed a trend towards an association of higher dietary GL in women with lower fecundability, which was significant among nulliparous women, in unadjusted models only. These effects attenuated after adjustment for other socio-demographic and lifestyle factors. No association was visible in multiparous women. Our findings, along with those from the Nurses’ Health Study, suggest the possibility of effect measure modification by parity in the relationship between dietary GI and GL and fecundability in women [11]. We hypothesize that metabolic responses to dietary carbohydrates may differ between nulliparous and multiparous women, likely due to hormonal and physiological distinctions associated with earlier pregnancies, potentially mitigating effects of dietary carbohydrates on fertility. Additionally, nulliparous women have been shown to be at higher risk of adverse birth outcomes, underscoring the importance of understanding these dietary associations in different reproductive populations [45].

To date, there has been a notable gap in research regarding the influence of dietary GI or GL in men on a couple’s time to pregnancy or subfertility risk. Linked to high dietary GI and GL, high male intake of sugar-sweetened beverages (≥ 7 beverages per week) was associated with reduced fecundability among couples planning a pregnancy, participating in the PRESTO cohort [46]. A case–control study including 552 asthenozoospermia cases, a condition of reduced sperm motility, and 585 normozoospermia controls found a positive association between the carbohydrate quality index and asthenozoospermia [47]. The carbohydrate quality index is a relatively new index to evaluate carbohydrate quality, which is calculated based on the ratio of solid carbohydrates to total carbohydrates, dietary fiber intake, the GI, and the ratio of whole grains to total grains. However, translation to other populations and clinical practice is challenging as considerable variation in how the carbohydrate quality index is defined across studies still occurs [48]. Our study specifically focused on GI and GL due to their well-established roles in influencing blood glucose, insulin regulation, and metabolic health, which are central to both sperm quality and ovulatory function. Previous small-scale studies in highly selected and at-risk populations have also suggested that paternal dietary GI and GL may affect sperm quality and function [14, 15]. Our study uniquely addressed both male and female dietary intake, allowing us to study the independent and combined effects of their dietary intake on time to pregnancy and fertility. Most previous cohort studies focused solely on male or female dietary GI or GL, and the association with fecundability [12]. Clearly, both parents play crucial roles in the underlying biological pathways influencing fertility, underscoring the importance of preconception interventions that address the health and dietary factors of both parents. Our study revealed that higher dietary GI and GL in men were associated with reduced fecundability, after adjusting for dietary GI or GL of the female partners and socio-demographic and lifestyle factors. When considering GI clinical categories, both women and men adhering to a low GI diet tended to be associated with increased fecundability and a lower subfertility risk, as compared to both partners adhering to a normal GI diet, with the effect primarily driven by the dietary GI category of the men. Thus, our study provides novel insights by demonstrating an association between higher dietary GI and GL with reduced fecundability in men, but not clearly in women. No significant associations were present with subfertility risk, possibly due to less power. These findings underscore the critical role of the dietary habits of both partners in optimizing reproductive health. The GI and GL are widely recognized and used in clinical settings, making our findings easily translatable to practical dietary recommendations, particularly for individuals our couples seeking to optimize their diet for fertility. Further research comparing the relative impacts of new carbohydrate quality indices with the GI and GL is essential to refine and expand these recommendations for clinical and preventive care.

The effects of high dietary GI and GL on fertility are thought to be mediated through insulin and its signaling pathway, which may adversely affect semen quality and ovulatory function. Elevated insulin levels from carbohydrate intake may increase free insulin-like growth factor 1 (IGF-1), its binding proteins, and sex hormone binding globulin (SHBG), creating an endocrine environment similar to that implicated in the clinical manifestations of PCOS [49]. Among women with PCOS, who often experience high insulin resistance, anovulation, and infertility, diets with a low GL have been shown to improve menstrual cycle regularity and insulin resistance [6]. Besides, hyperglycemia may directly contribute to excess formation of ROS and subsequent oxidative stress, which has been shown to influence spermatogenesis, as well as functionality of mature sperm and oocyte quality [7, 50, 51]. Another proposed mechanism suggests that impaired fertility is not directly due to increased carbohydrate intake, but rather to the reduction of natural fats, which are known to benefit ovulatory function and sperm quality [52].

While literature exploring the relationship between dietary carbohydrate quantity and quality of couple’s planning a pregnancy and fertility is expanding, discrepancies among findings of previous studies, which are predominantly observational in nature, and the limited evidence regarding the association between dietary carbohydrate quality and fertility in men weaken the strength of evidence for definitive recommendations for couples. Large randomized clinical trials and well-controlled feeding interventions during preconception should be conducted to further elucidate the effect of dietary GI and GL on fertility and to generate robust clinical guidelines. Ideally, these studies should include a larger variability in dietary GI and GL among multi-ethnic populations.

### Methodological considerations

To the best of our knowledge, this study is the first large-scale population-based cohort study from the preconception period onwards assessing dietary intake of couples during the periconception period and exploring associations of dietary GI and GL in both women and men with fertility. The non-response analysis revealed differences between women and men who completed the dietary questionnaire and those who did not participate. The selection towards a relatively healthy Dutch population may have affected the generalizability of our findings and might have led to reduced statistical power. Even though the FFQ is widely used for dietary assessment in observational studies, measurement of food intake by an FFQ may be affected by measurement error, recall bias, and/or reporting bias. Besides, the FFQ does not consider dietary supplement use. Subsequent calculation of the dietary GI and GL from the FFQ may further be affected by uncertainty induced by preparation of foods, mixed dishes, variations of food productions of time, or unavailability of specific food items. Accuracy of time to pregnancy duration may have been affected by the retrospectively answered questionnaires. To address this, time to pregnancy was reconfirmed during the first trimester visit to our research center. A validation study comparing prospectively measured time to pregnancy with time to pregnancy recalled during the first trimester of pregnancy reported perfect agreement between measures among 53% of their sample. Recall error was, on average, small (only 12% had a discrepancy of ≥ 2 months), with a median of 0 and a mean of − 0.11 months, indicating reasonable validity [53]. We collected dietary intake data at a single time point during the first trimester due to the design of our study, which we used as a proxy for periconception diet. Our approach was supported by existing literature, which shows that dietary patterns generally remain stable from the preconception period to early pregnancy [19, 20]. We consider the periconception period as most critical for this current study focused on fertility outcomes. Further studies are needed with repeated measurements of dietary intake at multiple time points from preconception throughout pregnancy, to better understand how parental dietary patterns evolve over time due to changing physiological and nutritional needs and impact the full course of pregnancy. Ideally, these studies should be conducted in diverse populations with varying dietary habits, aiming to determine the feasibility of a low GI and GL diet as a potential lifestyle intervention to improve couples fertility.

## Conclusions

Among couples in the general population, suboptimal carbohydrate intake may influence fertility in men, but not in women. Integrating dietary interventions for both partners into preconception care strategies may be a promising avenue to alleviate the growing worldwide burden of subfertility.

## Supplementary Information

Supplementary Fig. S1. Flowchart of the study population. Supplementary Fig. S2. Directed Acyclic Graph representing the pathways between the dietary glycemic index/load and fertility. Supplementary Table S1. Population characteristics according to periconception dietary glycemic index quartiles in women. Supplementary Table S2. Population characteristics according to periconception dietary glycemic index quartiles in men. Supplementary Table S3. Non-response analysis comparing population characteristics of women and men with and without dietary intake data available. Supplementary Table S4. Fecundability ratios for periconception dietary glycemic index and load quartiles. Supplementary Table S5. Associations of periconception dietary glycemic index and load quartiles with odds of subfertility. Supplementary Table S6. Associations of periconception dietary glycemic index clinical categories with fecundability and subfertility risk. Supplementary Table S7. Associations of periconception dietary glycemic index and load with fecundability and subfertility risk in women and men with overweight or obesity. Supplementary Table S8. Associations of periconception dietary glycemic index and load with fecundability and subfertility risk excluding women with pre-existing or gestational diabetes. Supplementary Table S9. Associations of periconception dietary glycemic index and load with fecundability and subfertility risk in women stratified for parity. Supplementary Information S1. Additional information on dietary intake data and processing

## Data Availability

The data that support the findings of this study are not openly available due to reasons of sensitivity and are available from the corresponding author upon reasonable request.

## Abbreviations

AI: Adequate intake
BMI: Body mass index
DM: Diabetes mellitus
EAR: Estimated average requirement
FR: Fecundability ratio
FFQ: Food frequency questionnaire
GI: Glycemic index
GL: Glycemic load
ICSI: Intracytoplasmic sperm injection
IGF-1: Insulin-like growth factor 1
IUI: Intrauterine insemination
IVF: In vitro fertilization
IR: Insulin resistance
PCOS: Polycystic ovary syndrome
PUFA: Poly-unsaturated fatty acids
ROS: Reactive oxygen species
SHBG: Sex hormone binding globulin

## References

[1] Sun H, Gong TT, Jiang YT, Zhang S, Zhao YH, Wu QJ. Global, regional, and national prevalence and disability-adjusted life-years for infertility in 195 countries and territories, 1990–2017: results from a global burden of disease study, 2017. Aging (Albany NY). 2019;11(23):10952–91. 31790362 10.18632/aging.102497 PMC6932903

[2] Cox CM, Thoma ME, Tchangalova N, Mburu G, Bornstein MJ, Johnson CL, Kiarie J. Infertility prevalence and the methods of estimation from 1990 to 2021: a systematic review and meta-analysis. Hum Reprod Open. 2022;2022(4):hoac051. 36483694 10.1093/hropen/hoac051 PMC9725182

[3] Taylor A. ABC of subfertility: extent of the problem. BMJ. 2003;327(7412):434–6. 12933733 10.1136/bmj.327.7412.434 PMC188498

[4] Practice Committee of American Society for Reproductive Medicine in collaboration with Society for Reproductive Endocrinology and Infertility. Optimizing natural fertility. Fertil Steril. 2008;90(5 Suppl):S1–6. 10.1016/j.fertnstert.2008.08.122 19007604

[5] Gaskins AJ, Chavarro JE. Diet and fertility: a review. Am J Obstet Gynecol. 2018;218(4):379–89. 28844822 10.1016/j.ajog.2017.08.010 PMC5826784

[6] Fontana R, Della TS. The deep correlation between energy metabolism and reproduction: a view on the effects of nutrition for women fertility. Nutrients. 2016;8(2):87. 26875986 10.3390/nu8020087 PMC4772050

[7] Łakoma K, Kukharuk O, Śliż D. The influence of metabolic factors and diet on fertility. Nutrients. 2023;15(5):1180. 10.3390/nu15051180 PMC10005661 36904180

[8] Jenkins DJ, Wolever TM, Taylor RH, Barker H, Fielden H, Baldwin JM, et al. Glycemic index of foods: a physiological basis for carbohydrate exchange. Am J Clin Nutr. 1981;34(3):362–6. 6259925 10.1093/ajcn/34.3.362

[9] Saadati N, Haidari F, Barati M, Nikbakht R, Mirmomeni G, Rahim F. The effect of low glycemic index diet on the reproductive and clinical profile in women with polycystic ovarian syndrome: a systematic review and meta-analysis. Heliyon. 2021;7(11): e08338. 34820542 10.1016/j.heliyon.2021.e08338 PMC8600081

[10] Becker GF, Passos EP, Moulin CC. Short-term effects of a hypocaloric diet with low glycemic index and low glycemic load on body adiposity, metabolic variables, ghrelin, leptin, and pregnancy rate in overweight and obese infertile women: a randomized controlled trial. Am J Clin Nutr. 2015;102(6):1365–72. 26561614 10.3945/ajcn.115.117200

[11] Chavarro JE, Rich-Edwards JW, Rosner BA, Willett WC. A prospective study of dietary carbohydrate quantity and quality in relation to risk of ovulatory infertility. Eur J Clin Nutr. 2009;63(1):78–86. 17882137 10.1038/sj.ejcn.1602904 PMC3066074

[12] Willis SK, Wise LA, Wesselink AK, Rothman KJ, Mikkelsen EM, Tucker KL, et al. Glycemic load, dietary fiber, and added sugar and fecundability in 2 preconception cohorts. Am J Clin Nutr. 2020;112(1):27–38. 31901163 10.1093/ajcn/nqz312 PMC7326597

[13] Willis SK, Wise LA, Laursen ASD, Wesselink AK, Mikkelsen EM, Tucker KL, et al. Glycemic load, dietary fiber, added sugar, and spontaneous abortion in two preconception cohorts. J Nutr. 2023;152(12):2818–26. 36057842 10.1093/jn/nxac202 PMC9839996

[14] Mateu-Fabregat J, Papandreou C, Gutierrez-Tordera L, Rojas M, Novau-Ferré N, Mostafa H, Bulló M. Dietary glycemic index and load and semen quality: a cross-sectional and prospective analysis within the FERTINUTS trial. World J Mens Health. 2024;42(4):881–9. 10.5534/wjmh.230328 PMC11439801 38772538

[15] Hosseini E, Khodavandloo M, Sabet SA, Mousavi SN. Relationship between dietary glycemic index and glycemic load and sperm-quality parameters in Iranian men: a cross-sectional study. BMC Nutr. 2024;10(1):34. 38409138 10.1186/s40795-024-00840-2 PMC10898108

[16] Kooijman MN, Kruithof CJ, van Duijn CM, Duijts L, Franco OH, van IMH, et al. The Generation R Study: design and cohort update 2017. Eur J Epidemiol. 2016;31(12):1243–64. 28070760 10.1007/s10654-016-0224-9 PMC5233749

[17] Beukers MH, Dekker LH, de Boer EJ, Perenboom CW, Meijboom S, Nicolaou M, et al. Development of the HELIUS food frequency questionnaires: ethnic-specific questionnaires to assess the diet of a multiethnic population in the Netherlands. Eur J Clin Nutr. 2015;69(5):579–84. 25226823 10.1038/ejcn.2014.180

[18] Rhee JJ, Sampson L, Cho E, Hughes MD, Hu FB, Willett WC. Comparison of methods to account for implausible reporting of energy intake in epidemiologic studies. Am J Epidemiol. 2015;181(4):225–33. 25656533 10.1093/aje/kwu308 PMC4325679

[19] Crozier SR, Robinson SM, Godfrey KM, Cooper C, Inskip HM. Women’s dietary patterns change little from before to during pregnancy. J Nutr. 2009;139(10):1956–63. 19710161 10.3945/jn.109.109579 PMC3113465

[20] Cucó G, Fernández-Ballart J, Sala J, Viladrich C, Iranzo R, Vila J, Arija V. Dietary patterns and associated lifestyles in preconception, pregnancy and postpartum. Eur J Clin Nutr. 2006;60(3):364–71. 16340954 10.1038/sj.ejcn.1602324

[21] Louie JC, Brand-Miller JC, Markovic TP, Ross GP, Moses RG. Glycemic index and pregnancy: a systematic literature review. J Nutr Metab. 2010;2010: 282464. 21253478 10.1155/2010/282464 PMC3022194

[22] Walsh JM, McGowan CA, Mahony R, Foley ME, McAuliffe FM. Low glycaemic index diet in pregnancy to prevent macrosomia (ROLO study): randomised control trial. BMJ. 2012;345: e5605. 22936795 10.1136/bmj.e5605 PMC3431285

[23] Salmerón J, Manson JE, Stampfer MJ, Colditz GA, Wing AL, Willett WC. Dietary fiber, glycemic load, and risk of non-insulin-dependent diabetes mellitus in women. JAMA. 1997;277(6):472–7. 9020271 10.1001/jama.1997.03540300040031

[24] Greenwood DC, Threapleton DE, Evans CE, Cleghorn CL, Nykjaer C, Woodhead C, Burley VJ. Glycemic index, glycemic load, carbohydrates, and type 2 diabetes: systematic review and dose-response meta-analysis of prospective studies. Diabetes Care. 2013;36(12):4166–71. 24265366 10.2337/dc13-0325 PMC3836142

[25] Aston LM, Jackson D, Monsheimer S, Whybrow S, Handjieva-Darlenska T, Kreutzer M, et al. Developing a methodology for assigning glycaemic index values to foods consumed across Europe. Obes Rev. 2010;11(1):92–100. 20653850 10.1111/j.1467-789X.2009.00690.x

[26] Okubo H, Crozier SR, Harvey NC, Godfrey KM, Inskip HM, Cooper C, Robinson SM. Maternal dietary glycemic index and glycemic load in early pregnancy are associated with offspring adiposity in childhood: the Southampton Women’s Survey. Am J Clin Nutr. 2014;100(2):676–83. 24944056 10.3945/ajcn.114.084905

[27] Wahab RJ, Scholing JM, Gaillard R. Maternal early pregnancy dietary glycemic index and load, fetal growth, and the risk of adverse birth outcomes. Eur J Nutr. 2021;60(3):1301–11. 32666314 10.1007/s00394-020-02327-9 PMC7987612

[28] Zhang R, Han S, Chen GC, Li ZN, Silva-Zolezzi I, Parés GV, et al. Effects of low-glycemic-index diets in pregnancy on maternal and newborn outcomes in pregnant women: a meta-analysis of randomized controlled trials. Eur J Nutr. 2018;57(1):167–77. 27612876 10.1007/s00394-016-1306-x

[29] Infertility workup for the women’s health specialist. ACOG Committee opinion, number 781. Obstet Gynecol. 2019;133(6):e377–84. 31135764 10.1097/AOG.0000000000003271

[30] Central Bureau for Statistics. Migrants in the Netherlands. Central Bureau for Statistics; 2003.

[31] Troe EJ, Raat H, Jaddoe VW, Hofman A, Looman CW, Moll HA, et al. Explaining differences in birthweight between ethnic populations. The Generation R Study Bjog. 2007;114(12):1557–65. 17903227 10.1111/j.1471-0528.2007.01508.x

[32] Health Council of the Netherlands. Dietary reference intakes: energy, proteins, fats and digestible carbohydrates. The Hague: Health Council of the Netherlands; 2001.

[33] Health Council of the Netherlands. Guideline for dietary fiber intake. The Hague: Health Council of the Netherlands; 2006.

[34] Health Council of the Netherlands. Dietary reference values for vitamins and minerals for adults. The Hague: Health Council of the Netherlands; 2018.

[35] Health Council of the Netherlands. Dietary reference values for protein. The Hague: Health Council of the Netherlands; 2021.

[36] VanderWeele TJ, Hernán MA, Robins JM. Causal directed acyclic graphs and the direction of unmeasured confounding bias. Epidemiology. 2008;19(5):720–8. 18633331 10.1097/EDE.0b013e3181810e29 PMC4242711

[37] Textor J, van der Zander B, Gilthorpe MS, Liskiewicz M, Ellison GT. Robust causal inference using directed acyclic graphs: the R package ‘dagitty.’ Int J Epidemiol. 2016;45(6):1887–94. 28089956 10.1093/ije/dyw341

[38] Rossi BV, Abusief M, Missmer SA. Modifiable risk factors and infertility: what are the connections? Am J Lifestyle Med. 2014;10(4):220–31. 27594813 10.1177/1559827614558020 PMC5007064

[39] Steiner AZ, Jukic AM. Impact of female age and nulligravidity on fecundity in an older reproductive age cohort. Fertil Steril. 2016;105(6):1584-8 e1. 26953733 10.1016/j.fertnstert.2016.02.028 PMC4893975

[40] Lane M, Robker RL, Robertson SA. Parenting from before conception. Science. 2014;345(6198):756–60. 25124428 10.1126/science.1254400

[41] Stephenson J, Heslehurst N, Hall J, Schoenaker D, Hutchinson J, Cade JE, et al. Before the beginning: nutrition and lifestyle in the preconception period and its importance for future health. Lancet. 2018;391(10132):1830–41. 29673873 10.1016/S0140-6736(18)30311-8 PMC6075697

[42] Caut C, Leach M, Steel A. Dietary guideline adherence during preconception and pregnancy: a systematic review. Matern Child Nutr. 2020;16(2): e12916. 31793249 10.1111/mcn.12916 PMC7083492

[43] St-Laurent A, Savard C, Plante AS, Gagnon M, Robitaille J, Lemieux S, et al. Health-related preconception factors: adherence to guidelines and associations with weight status. J Acad Nutr Diet. 2022;122(10):1911–21. 35367418 10.1016/j.jand.2022.03.012

[44] Grieger JA, Grzeskowiak LE, Bianco-Miotto T, Jankovic-Karasoulos T, Moran LJ, Wilson RL, et al. Pre-pregnancy fast food and fruit intake is associated with time to pregnancy. Hum Reprod. 2018;33(6):1063–70. 29733398 10.1093/humrep/dey079

[45] Miranda ML, Edwards SE, Myers ER. Adverse birth outcomes among nulliparous vs. multiparous women. Public Health Rep. 2011;126(6):797–805. 10.1177/003335491112600605 PMC3185315 22043095

[46] Hatch EE, Wesselink AK, Hahn KA, Michiel JJ, Mikkelsen EM, Sorensen HT, et al. Intake of sugar-sweetened beverages and fecundability in a North American preconception cohort. Epidemiology. 2018;29(3):369–78. 29384791 10.1097/EDE.0000000000000812 PMC5882510

[47] Li XY, Zhang YX, Wang XB, Nan YX, Wang DD, Sun MH, et al. Associations between dietary macronutrient quality and asthenozoospermia risk: a hospital-based case-control study. Food Funct. 2024;15(12):6383–94. 38819120 10.1039/d4fo01234h

[48] Maghoul A, Khonsari NM, Asadi S, Abdar ZE, Ejtahed HS, Qorbani M. Dietary carbohydrate quality index and cardio-metabolic risk factors. Int J Vitam Nutr Res. 2024;94(5–6):377–93. 38009678 10.1024/0300-9831/a000794

[49] Rojas J, Chávez M, Olivar L, Rojas M, Morillo J, Mejías J, et al. Polycystic ovary syndrome, insulin resistance, and obesity: navigating the pathophysiologic labyrinth. Int J Reprod Med. 2014;2014: 719050. 25763405 10.1155/2014/719050 PMC4334071

[50] Maresch CC, Stute DC, Alves MG, Oliveira PF, de Kretser DM, Linn T. Diabetes-induced hyperglycemia impairs male reproductive function: a systematic review. Hum Reprod Update. 2018;24(1):86–105. 29136166 10.1093/humupd/dmx033

[51] Martins AD, Majzoub A, Agawal A. Metabolic syndrome and male fertility. World J Mens Health. 2019;37(2):113–27. 30350486 10.5534/wjmh.180055 PMC6479081

[52] Wathes DC, Abayasekara DR, Aitken RJ. Polyunsaturated fatty acids in male and female reproduction. Biol Reprod. 2007;77(2):190–201. 17442851 10.1095/biolreprod.107.060558

[53] Radin RG, Rothman KJ, Hatch EE, Mikkelsen EM, Sorensen HT, Riis AH, et al. Maternal recall error in retrospectively reported time-to-pregnancy: an assessment and bias analysis. Paediatr Perinat Epidemiol. 2015;29(6):576–88. 26443987 10.1111/ppe.12245 PMC4651209

