# Supplementary material for: Associations of periconception dietary glycemic index and load with fertility in women and men: a study among couples in the general population
Source: BMC Med. 2024 Oct 29;22:499. doi: 10.1186/s12916-024-03718-z (PMC11520767; doi:10.1186/s12916-024-03718-z)
Supplement: Supplementary file 1 — Supplementary Fig. S1. Flowchart of the study population. Supplementary Fig. S2. Directed Acyclic Graph representing the pathways between the dietary glycemic index/load and fertility. Supplementary Table S1. Population characteristics according to periconception dietary glycemic index quartiles in women. Supplementary Table S2. Population characteristics according to periconception dietary glycemic index quartiles in men. Supplementary Table S3. Non-response analysis comparing population characteristics of women and men with and without dietary intake data available. Supplementary Table S4. Fecundability ratios for periconception dietary glycemic index and load quartiles. Supplementary Table S5. Associations of periconception dietary glycemic index and load quartiles with odds of subfertility. Supplementary Table S6. Associations of periconception dietary glycemic index clinical categories with fecundability and subfertility risk. Supplementary Table S7. Associations of periconception dietary glycemic index and load with fecundability and subfertility risk in women and men with overweight or obesity. Supplementary Table S8. Associations of periconception dietary glycemic index and load with fecundability and subfertility risk excluding women with pre-existing or gestational diabetes. Supplementary Table S9. Associations of periconception dietary glycemic index and load with fecundability and subfertility risk in women stratified for parity. Supplementary Information S1. Additional information on dietary intake data and processing [file 12916_2024_3718_MOESM1_ESM.docx]

**Additional file 1**

**Associations of periconception dietary glycemic index and load with fertility in women and men: a study among couples in the general population**

Mireille C. Schipper^1,2^, Aline J. Boxem MD^1,2^, Sophia M. Blaauwendraad MD^1,2^, Annemarie G.M.G.J. Mulders MD PhD^3^, Vincent W.V. Jaddoe MD PhD^1,2^, Romy Gaillard MD PhD^1,2^.

1.The Generation R Study Group, Erasmus MC, University Medical Center, Rotterdam, the Netherlands.

2.Department of Pediatrics, Sophia’s Children’s Hospital, Erasmus MC, University Medical Center, Rotterdam, the Netherlands.

3.Department of Obstetrics and Gynecology, Erasmus MC, University Medical Center, Rotterdam, the Netherlands.

Corresponding author**:** Romy Gaillard, The Generation R Study Group, Erasmus University Medical Center, PO Box 2040, 3000 CA Rotterdam, the Netherlands ([r.gaillard@erasmusmc.nl)](mailto:r.gaillard@erasmusmc.nl)). Telephone number: 0031 10 704 3405.

**CONTENTS**

**Supplementary Figure S1.** Flowchart of the study population

**Supplementary Figure S2**. Directed Acyclic Graph representing the pathways between the dietary glycemic index/load and fertility

**Supplementary Table S1**. Population characteristics according to periconception dietary glycemic index quartiles in women

**Supplementary Table S2**. Population characteristics according to periconception dietary glycemic index quartiles in men

**Supplementary Table S3**. Non-response analysis comparing population characteristics of women and men with and without dietary intake data available

**Supplementary Table S4**. Fecundability ratios for periconception dietary glycemic index and load quartiles

**Supplementary Table S5**. Associations of periconception dietary glycemic index and load quartiles with odds of subfertility

**Supplementary Table S6**. Associations of periconception dietary glycemic index clinical categories with fecundability and subfertility risk

**Supplementary Table S7**. Associations of periconception dietary glycemic index and load with fecundability and subfertility risk in women and men with overweight or obesity

**Supplementary Table S8**. Associations of periconception dietary glycemic index and load with fecundability and subfertility risk excluding women with pre-existing or gestational diabetes

**Supplementary Table S9**. Associations of periconception dietary glycemic index and load with fecundability and subfertility risk in women stratified for parity

**Supplementary Information S1.** Additional information on dietary intake data and processing

**Supplementary Figure S1**. Flowchart of the study population


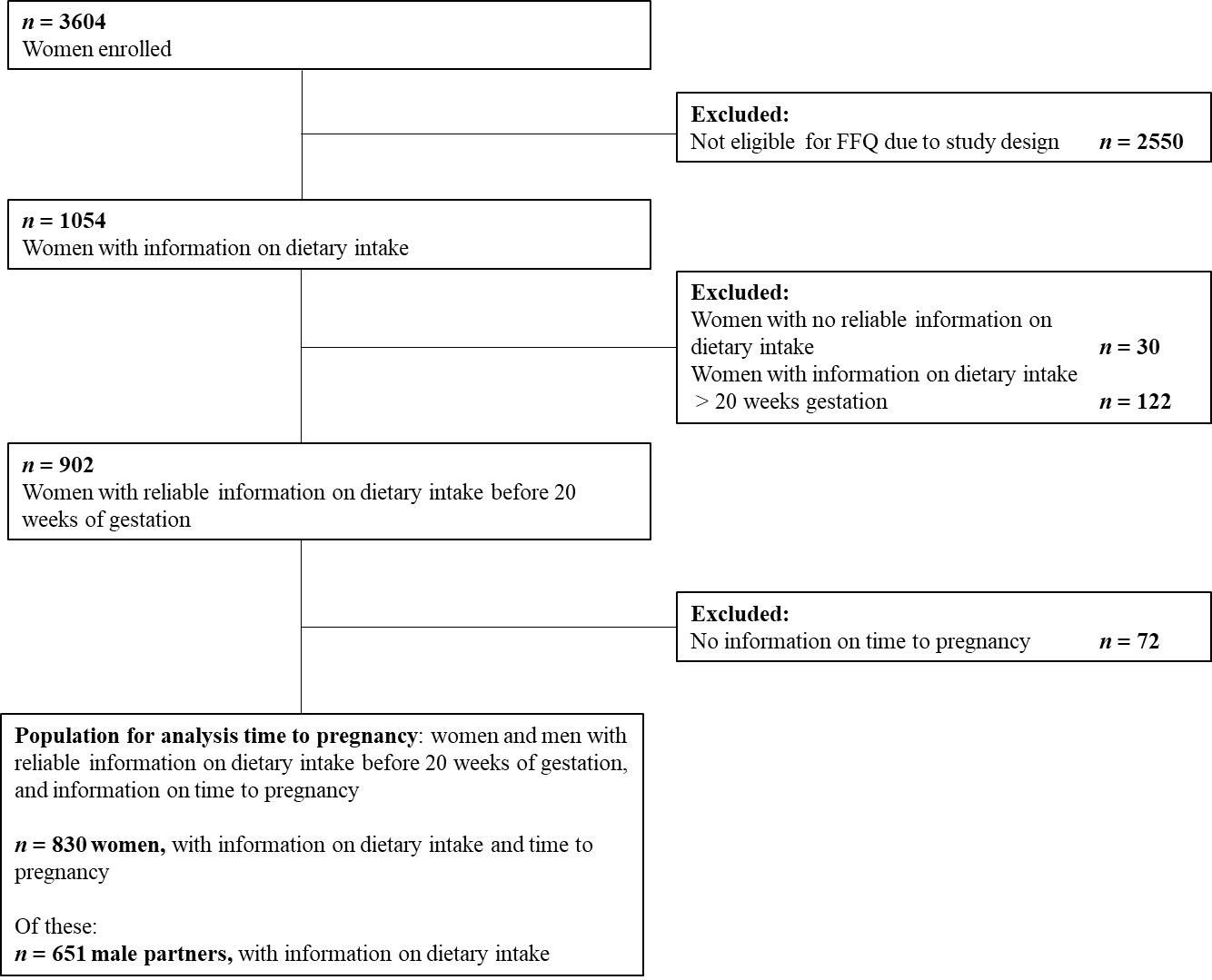


**Supplementary Figure S2.** Directed Acyclic Graph representing the pathways between the dietary glycemic index/load and fertility


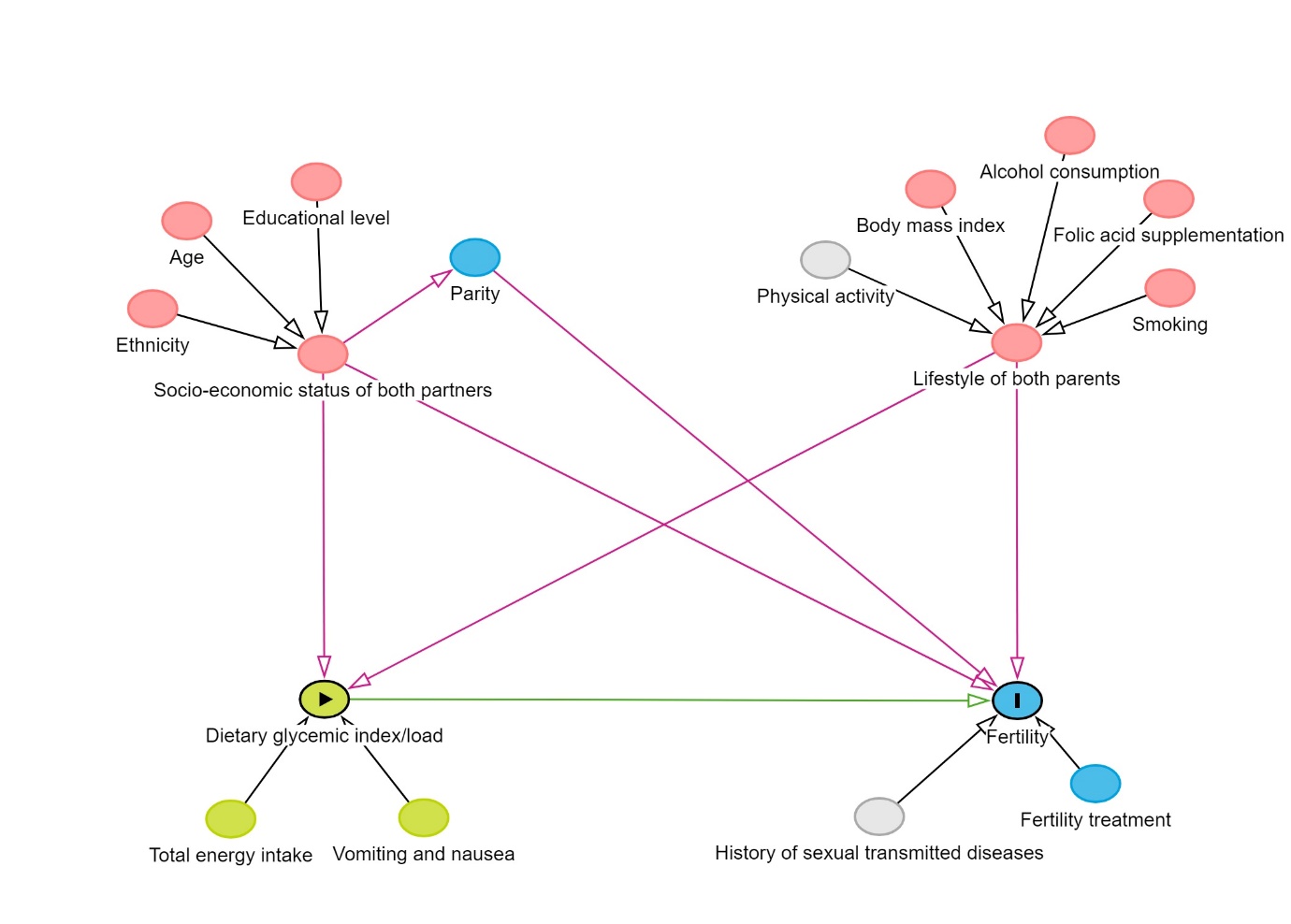


**Supplementary Table S1.** Population characteristics according to periconception dietary glycemic index quartiles in women

|  | **Total group**  *n* = 830 | **Glycemic index quartile 1**  *n* = 208 | **Glycemic index quartile 2**  *n* = 207 | **Glycemic index quartile 3**  *n* = 207 | **Glycemic index quartile 4**    *n* = 208 | **P-value** |
| --- | --- | --- | --- | --- | --- | --- |
| ***Population characteristics*** |  |  |  |  |  |  |
| Age at dietary intake assessment, mean (SD), years | 32.1 (3.9) | 33.4 (3.6) | 32.6 (3.8) | 31.5 (3.7) | 31.2 (4.1) | <0.001 |
| Gestational age at dietary intake assessment, median, (95% range), weeks | 12.4 (10.9, 18.4) | 12.5 (10.9, 19.2) | 12.4 (10.9, 18.0) | 12.4 (10.9, 17.9) | 12.4 (11.0, 19.1) | 0.635 |
| Ethnicity, % (*n*) |  |  |  |  |  | 0.010 |
| Dutch | 69.0 (570) | 71.2 (148) | 70.0 (145) | 69.6 (142) | 65.2 (135) |  |
| European | 10.0 (83) | 13.5 (28) | 9.7 (20) | 11.3 (23) | 5.8 (12) |  |
| Non-European | 20.9 (173) | 15.4 (32) | 20.3 (42) | 19.1 (39) | 29.0 (60) |  |
| Education level, high, % (*n*) | 82.5 (675) | 92.3 (191) | 84.5 (174) | 84.3 (172) | 68.7 (138) | <0.001 |
| (Pre-pregnancy) body mass index, median (IQR), kg/m^2^ | 23.0 (21.1, 25.3) | 22.4 (20.8, 24.1) | 22.7 (20.9, 24.7) | 23.3 (21.1, 26.3) | 23.7 (21.9, 26.1) | <0.001 |
| Overweight/obesity, % (*n*) | 27.6 (222) | 19.0 (38) | 21.7 (44) | 35.1 (71) | 34.5 (69) | <0.001 |
| Smoking before pregnancy, % (*n*) | 41.7 (315) | 39.1 (75) | 44.0 (80) | 41.7 (80) | 42.1 (80) | 0.815 |
| Alcohol use before pregnancy, % (*n*) | 85.1 (701) | 87.0 (180) | 87.3 (179) | 84.5 (175) | 81.5 (167) | 0.316 |
| Drug use before pregnancy, % (*n*) | 8.7 (72) | 10.2 (21) | 7.7 (16) | 7.7 (16) | 9.2 (19) | 0.770 |
| Periconception folic acid supplement use, % (*n*) | 99.5 (804) | 99.0 (201) | 100.0 (202) | 100.0 (204) | 99.0 (197) | 0.247 |
| Parity, nulliparous, % (*n*) | 72.4 (594) | 72.8 (150) | 70.5 (146) | 78.2 (161) | 67.8 (137) | 0.116 |
| Pre-existing or gestational diabetes, % (*n*) | 6.8 (56) | 5.3 (11) | 10.2 (21) | 3.9 (8) | 7.8 (16) | 0.053 |
| Daily nausea and vomiting during early pregnancy, % (*n*) | 2.5 (20) | 1.0 (2) | 3.1 (6) | 3.0 (6) | 3.0 (6) | 0.458 |
| Previous miscarriage, % (*n*) | 19.1 (142) | 20.5 (39) | 16.1 (29) | 16.8 (32) | 22.7 (42) | 0.324 |
| Glycemic index, mean (SD) | 56.2 (3.5) | 51.9 (1.6) | 54.9 (0.6) | 57.2 (0.7) | 60.8 (1.6) | <0.001 |
| Glycemic load, mean (SD) | 141.4 (67.4) | 100.3 (30.9) | 117.7 (34.0) | 141.4 (46.0) | 206.2 (86.6) | <0.001 |
|  |  |  |  |  |  |  |
| ***Outcome characteristics*** |  |  |  |  |  |  |
| Time to pregnancy, median (IQR), months | 4.8 (1.2, 16.4) | 4.6 (0.8, 15.5) | 5.8 (1.3, 18.1) | 4.7 (1.2, 15.9) | 4.8 (1.5, 15.8) | 0.598 |
| Time to pregnancy ≥ 12 months (subfertility), % (*n*) | 30.6 (254) | 29.3 (61) | 32.9 (68) | 29.5 (61) | 30.8 (64) | 0.854 |
| Pregnancy results of fertility treatment, % (*n*) | 13.2 (108) | 12.8 (26) | 14.7 (30) | 13.3 (27) | 12.1 (25) | 0.889 |
| P-values calculated using one-way ANOVA tests, Kruskal Wallis tests, Chi-square tests or Fisher’s Exact tests. | | | | | | |

**Supplementary Table S2.** Population characteristics according to periconception dietary glycemic index quartiles in men

|  | **Total group**  *n* = 651 | **Glycemic index quartile 1**  *n =* 163 | **Glycemic index quartile 2**  *n =* 163 | **Glycemic index quartile 3**  *n* = 162 | **Glycemic index quartile 4**  *n* = 163 | **P-value** |
| --- | --- | --- | --- | --- | --- | --- |
| ***Population characteristics*** |  |  |  |  |  |  |
| Age at dietary intake assessment, mean (SD), years | 34.2 (5.0) | 34.7 (4.7) | 34.2 (4.5) | 34.2 (5.0) | 33.5 (5.8) | 0.076 |
| Ethnicity, % (*n*) |  |  |  |  |  | 0.085 |
| Dutch | 74.8 (485) | 79.6 (129) | 77.2 (125) | 72.8 (118) | 69.8 (113) |  |
| European | 7.9 (51) | 9.9 (16) | 6.2 (10) | 8.6 (14) | 6.8 (11) |  |
| Non-European | 17.3 (112) | 10.5 (17) | 16.7 (27) | 18.5 (30) | 23.5 (38) |  |
| Education level, high, % (*n*) | 75.0 (487) | 87.7 (142) | 80.9 (131) | 74.1 (120) | 57.7 (94) | <0.001 |
| Body mass index, median (IQR), kg/m^2^ | 24.6 (22.7, 26.8) | 24.2 (22.2, 26.1) | 24.4 (22.7, 26.0) | 24.7 (23.1, 26.6) | 25.3 (23.0, 27.8) | 0.002 |
| Overweight/obesity, % (*n*) | 44.3 (286) | 37.3 (60) | 39.5 (64) | 46.9 (75) | 53.7 (87) | 0.012 |
| Smoking before pregnancy, % (*n*) | 48.4 (313) | 41.0 (66) | 43.8 (71) | 51.6 (83) | 57.1 (93) | 0.015 |
| Alcohol use before pregnancy, % (*n*) | 92.1 (596) | 93.8 (150) | 94.5 (154) | 91.3 (147) | 89.0 (145) | 0.238 |
| Diabetes Mellitus, % (*n*) | 0.9 (6) | 0.6 (1) | 0.6 (1) | 1.9 (3) | 0.6 (1) | 0.673 |
| Glycemic index, mean (SD) | 56.8 (3.2) | 52.9 (1.3) | 55.4 (0.6) | 57.7 (0.7) | 61.1 (1.6) | <0.001 |
| Glycemic load, mean (SD) | 156.7 (75.4) | 124.2 (34.1) | 134.0 (37.9) | 151.0 (48.5) | 217.7 (111.9) | <0.001 |
| P-values calculated using one-way ANOVA tests, Kruskal Wallis tests, Chi-square tests or Fisher’s Exact tests. | | | | | | |

**Supplementary Table S3.** Non-response analysis comparing population characteristics of women and men with and without dietary intake data available

|  | **Women without dietary intake data**  *n* = 431 | **Women with dietary intake data**    *n* = 1054 | **P-value** | **Men without dietary intake data**  *n* = 595 | **Men with dietary intake data**  *n* = 902 | **P-value** |
| --- | --- | --- | --- | --- | --- | --- |
| Ethnicity, % (*n*) |  |  | <0.001 |  |  | <0.001 |
| Dutch | 44.0 (155) | 67.1 (696) |  | 44.0 (209) | 72.8 (647) |  |
| European | 12.8 (45) | 9.7 (101) |  | 10.9 (52) | 8.0 (71) |  |
| Non-European | 43.2 (152) | 23.2 (241) |  | 45.1 (214) | 19.2 (171) |  |
| Education level, high, % (*n*) | 52.4 (183) | 79.4 (819) | <0.001 | 47.5 (225) | 72.2 (644) | <0.001 |
| Parity, nulliparous, % (*n*) | 54.7 (152) | 69.9 (719) | <0.001 | na | na |  |
| Pre-pregnancy body mass index, median (IQR), kg/m^2^ | 24.3 (21.7, 28.3) | 23.1 (21.1, 25.6) | <0.001 | 25.5 (23.2, 28.6) | 24.7 (22.8, 26.9) | <0.001 |
| Overweight/obesity, % (*n*) | 43.5 (114) | 29.2 (290) | <0.001 | 58.2 (210) | 45.7 (408) | <0.001 |
| Periconception folic acid supplement use, % (*n*) | 98.3 (231) | 99.5 (993) | 0.073 | na | na |  |
| Smoking before pregnancy, yes, % (*n*) | 52.6 (131) | 44.0 (403) | 0.016 | 52.9 (198) | 47.9% (421) | 0.106 |
| Alcohol before pregnancy, yes % (*n*) | 70.8 (189) | 82.4 (845) | <0.001 | 82.7 (315) | 90.8 (797) | <0.001 |
| Drug use before pregnancy, yes % (*n*) | 8.2 (26) | 9.8 (102) | 0.4 | na | na |  |
| P-values calculated using *t*-tests, Mann-Whitney *U*-tests, Chi-square tests or Fisher’s Exact tests. | | | | | | |

**Supplementary Table S4**. Fecundability ratios for periconception dietary glycemic index and load quartiles

|  | Fecundability ratio (95%) | | |
| --- | --- | --- | --- |
|  |  |  |  |
|  |  | Unadjusted model | Confounder model |
| ***Women*** | |  |  |
| Glycemic index | |  |  |
| Quartile 1 | | *Ref* | *Ref* |
| Quartile 2 | | 0.89 (0.73, 1.08) | 0.82 (0.67, 1.00) |
| Quartile 3 | | 0.98 (0.80, 1.18) | 0.89 (0.73, 1.08) |
| Quartile 4 | | 0.96 (0.79, 1.16) | 0.87 (0.71, 1.07) |
| Glycemic load | |  |  |
| Quartile 1 | | *Ref* | *Ref* |
| Quartile 2 | | 0.86 (0.71, 1.04) | 0.92 (0.75, 1.13) |
| Quartile 3 | | 0.85 (0.70, 1.03) | 0.86 (0.68, 1.08) |
| Quartile 4 | | 0.86 (0.71, 1.04) | 0.89 (0.68, 1.16) |
| ***Men*** | |  |  |
| Glycemic index | |  |  |
| Quartile 1 | | *Ref* | *Ref* |
| Quartile 2 | | 1.01 (0.82, 1.26) | 1.01 (0.81, 1.26) |
| Quartile 3 | | 0.80 (0.64, 1.00) | 0.82 (0.65, 1.02) |
| Quartile 4 | | 0.91 (0.73, 1.13) | 0.92 (0.73, 1.16) |
| Glycemic load | |  |  |
| Quartile 1 | | *Ref* | *Ref* |
| Quartile 2 | | 0.94 (0.76, 1.17) | 1.00 (0.79, 1.27) |
| Quartile 3 | | 0.93 (0.75, 1.16) | 0.92 (0.71, 1.19) |
| Quartile 4 | | 0.98 (0.78, 1.21) | 0.95 (0.71, 1.29) |
| Values represent the fecundability per quartile increase in the dietary glycemic index or load for women and men, as compared to the lowest quartile serving as the reference category. Fecundability represents the probability of conceiving within one month (28 days). Models were analysed using Cox proportional hazards models. Fecundability ratios were derived from the hazard ratios of the Cox proportional hazards models.  Confounder models in women included: age, ethnicity, educational level, alcohol use, smoking, pre-pregnancy body mass index, parity, and total energy intake.  Confounder models in men included: age, ethnicity, educational level, alcohol use, smoking, body mass index, and total energy intake. | | | |

**Supplementary Table S5**. Associations of periconception dietary glycemic index and load quartiles with odds of subfertility

|  | Odds ratio (95%) | |
| --- | --- | --- |
|  |  |  |
|  | Unadjusted model | Confounder model |
| ***Women*** |  | |
| Glycemic index |  | |
| Quartile 1 | *Ref* | *Ref* |
| Quartile 2 | 1.18 (0.78, 1.79) | 1.23 (0.79, 1.91) |
| Quartile 3 | 1.01 (0.66, 1.54) | 1.17 (0.75, 1.84) |
| Quartile 4 | 1.07 (0.70, 1.63) | 1.15 (0.72, 1.82) |
| Glycemic load |  | |
| Quartile 1 | *Ref* | *Ref* |
| Quartile 2 | 1.47 (0.96, 2.27) | 1.49 (0.93, 2.40) |
| Quartile 3 | 1.51 (0.98, 2.32) | 1.62 (0.96, 2.74) |
| Quartile 4 | 1.50 (0.98, 2.30) | 1.62 (0.90, 2.92) |
| ***Men*** |  |  |
| Glycemic index |  | |
| Quartile 1 | *Ref* | *Ref* |
| Quartile 2 | 0.97 (0.58, 1.61) | 0.98 (0.58, 1.66) |
| Quartile 3 | 1.50 (0.92, 2.44) | 1.51 (0.91, 2.50) |
| Quartile 4 | 1.36 (0.84, 2.22) | 1.33 (0.79, 2.25) |
| Glycemic load |  | |
| Quartile 1 | *Ref* | *Ref* |
| Quartile 2 | 1.17 (0.72, 1.91) | 1.11 (0.65, 1.92) |
| Quartile 3 | 1.04 (0.63, 1.71) | 1.09 (0.60, 2.00) |
| Quartile 4 | 1.27 (0.79, 2.07) | 1.31 (0.68, 2.53) |
| Values represent the odds of subfertility (≥ 12 months to conceive), associated with each quartile increase in glycemic index or glycemic load in women and men, with the lowest quartile serving as the reference category. Models were analysed using logistic regression models.  Confounder models in women included: age, ethnicity, educational level, alcohol use, smoking, pre-pregnancy body mass index, parity, and total energy intake.  Confounder models in men included: age, ethnicity, educational level, alcohol use, smoking, body mass index, and total energy intake. | | |

**Supplementary Table S6**. Associations of periconception dietary glycemic index clinical categories with fecundability and subfertility risk

|  |  | ^A^Fecundability ratio (95% CI) | Subfertility  ^B^Odds ratio  (95% CI) | |
| --- | --- | --- | --- | --- |
|  |  |  |  |  |
|  | *n* | Confounder model | Unadjusted model | Confounder model |
| ***Women*** | 830 |  |  |  |
| Normal GI diet |  | *Ref* | *Ref* | *Ref* |
| Low GI diet |  | 1.09 (0.94, 1.26) | 1.01 (0.75, 1.37) | 0.96 (0.69, 1.34) |
| ***Men*** | 651 |  |  |  |
| Normal GI diet |  | *Ref* | *Ref* | *Ref* |
| Low GI diet |  | 1.08 (0.91, 1.29) | 0.80 (0.55, 1.16) | 0.82 (0.55, 1.21) |
|  | |  |  |  |
| ***Combined clinical categories*** | 651 |  |  |  |
| Both partners normal GI diet |  | *Ref* | *Ref* | *Ref* |
| Women low GI diet & men normal GI diet |  | 1.07 (0.87, 1.32) | 1.06 (0.68, 1.63) | 1.05 (0.65, 1.70) |
| Women normal GI diet & Men low GI diet |  | 1.03 (0.80, 1.31) | 0.92 (0.54, 1.53) | 1.00 (0.56, 1.77) |
| Both partners low GI diet |  | 1.25 (0.99, 1.58) | 0.74 (0.45, 1.21) | 0.73 (0.42, 1.26) |
| **A)** Values represent the fecundability when compared to the reference category. Fecundability represents the probability of conceiving within one month (28 days). Models were analyzed using Cox proportional hazard models. Fecundability ratios were derived from the hazard ratios of the Cox proportional hazards models. **B)** Values represent the odds of subfertility (≥ 12 months to conceive), for women and men, as compared to the reference category. Models were analysed using logistic regression models. Combined clinical categories combined the glycemic index clinical category of both partners.  Confounder models in women included: age, ethnicity, educational level, alcohol use, smoking, pre-pregnancy body mass index, parity, and total energy intake.  Cofounder models in men included: age, ethnicity, educational level, alcohol use, smoking, body mass index, and total energy intake.  Combined confounder models included all confounders listed for both women and men. | | | | |

**Supplementary Table S7.** Associations of periconception dietary glycemic index and load with fecundability and subfertility risk in women and men with overweight or obesity

|  |  | | ^A^Fecundability ratio (95% CI) | | Subfertility  ^B^Odds ratio (95% CI) | |
| --- | --- | --- | --- | --- | --- | --- |
|  |  |  |  |  |  |  |
|  |  | *n* | Unadjusted model | Confounder model | Unadjusted model | Confounder model |
| Glycemic index (SDS) | Women | 222 | 1.07 (0.95, 1.21) | 1.04 (0.91, 1.19) | 0.85 (0.64, 1.13) | 0.90 (0.66, 1.23) |
|  | Men | 286 | 0.93 (0.82, 1.04) | 0.91 (0.80, 1.03) | 1.16 (0.90, 1.50) | 1.19 (0.90, 1.58) |
| Glycemic load (SDS) | Women | 222 | 0.96 (0.84, 1.11) | 0.98 (0.83, 1.17) | 1.07 (0.81, 1.40) | 1.06 (0.75. 1.50) |
|  | Men | 286 | 0.99 (0.88, 1.11) | 1.01 (0.87, 1.17) | 1.02 (0.80, 1.29) | 0.98 (0.73, 1.31) |
| **A**) Values represent the fecundability per SDS (standard deviation score) increase the dietary glycemic index or load exclusively in women and men with overweight or obesity. Fecundability represents the probability of conceiving within one month (28 days). Models were analysed using Cox proportional hazard models. Fecundability ratios were derived from the hazard ratios of the Cox proportional hazard models. **B**) Values represent the odds of subfertility (≥ 12 months to conceive) per SDS increase in the glycemic index or load exclusively in women and men with overweight or obesity. Models were analysed using logistic regression models.  Confounder models in women included: age, ethnicity, educational level, alcohol use, smoking, parity, and total energy intake.  Confounder models in men included: age, ethnicity, educational level, alcohol use, smoking, and total energy intake. | | | | | | |

**Supplementary Table S8.** Associations of periconception dietary glycemic index and load with fecundability and subfertility risk excluding women and men with pre-existing or gestational diabetes

|  |  |  | ^A^Fecundability ratio (95% CI) | | Subfertility  ^B^Odds ratio (95% CI) | |
| --- | --- | --- | --- | --- | --- | --- |
|  |  |  |  |  |  |  |
|  |  | *n* | Unadjusted model | Confounder model | Unadjusted model | Confounder model |
| Glycemic index (SDS) | Women | 767 | 1.01 (0.94, 1.08) | 0.96 (0.89, 1.04) | 0.99 (0.85, 1.16) | 1.03 (0.87, 1.22) |
|  | Men | 626 | 0.93 (0.86, 1.01) | 0.91 (0.84, 0.99)* | 1.16 (0.98, 1.39) | 1.19 (0.99, 1.44) |
| Glycemic load (SDS) | Women | 767 | 0.95 (0.88, 1.02) | 0.95 (0.86, 1.04) | 1.13 (0.97, 1.31) | 1.13 (0.93, 1.37) |
|  | Men | 626 | 0.98 (0.91, 1.06) | 0.92 (0.83, 1.01) | 1.09 (0.92, 1.29) | 1.16 (0.94, 1.44) |
| **A)** Values represent the fecundability per SDS (standard deviation score) increase in the dietary glycemic index or load excluding women and men with pre-existing or gestational diabetes (in women). Fecundability represents the probability of conceiving within one month (28 days). Models were analysed using Cox proportional hazard models. Fecundability ratios were derived from the hazard ratios of the Cox proportional hazard models. **B**) Values represent the odds of subfertility (≥ 12 months to conceive), per SDS increase in the glycemic index or load, excluding women and men with pre-existing or gestational diabetes (in women). Models were analysed using logistic regression models.  Confounder models in women included: age, ethnicity, educational level, alcohol use, smoking, pre-pregnancy body mass index, parity, and total energy intake.  Confounder models in men included: age, ethnicity, educational level, alcohol use, smoking, body mass index, and total energy intake. | | | | | | |

**Supplementary Table S9.** Associations of periconception dietary glycemic index and load with fecundability and subfertility risk in women stratified for parity

|  |  | ^A^Fecundability ratio (95% CI) | | | Subfertility  ^B^Odds ratio (95% CI) | |
| --- | --- | --- | --- | --- | --- | --- |
|  |  |  |  |  |  |  |
|  |  | *n* | Unadjusted model | Confounder model | Unadjusted model | Confounder model |
| Glycemic index (SDS) | Nulliparous | 594 | 0.99 (0.92, 1.08) | 0.95 (0.87, 1.04) | 1.04 (0.87, 1.26) | 1.10 (0.90, 1.34) |
|  | Multiparous | 227 | 1.05 (0.93, 1.19) | 0.96 (0.83, 1.12) | 0.90 (0.69, 1.16) | 0.93 (0.68, 1.26) |
| Glycemic load (SDS) | Nulliparous | 594 | 0.92 (0.85, 1.00)* | 0.92 (0.82, 1.02) | 1.22 (1.03, 1.45) | 1.24 (0.99, 1.56) |
|  | Multiparous | 227 | 1.01 (0.88, 1.16) | 0.97 (0.81, 1.15) | 0.93 (0.69, 1.22) | 0.97 (0.68, 1.38) |
| **A)** Values represent the fecundability per SDS (standard deviation score) increase in dietary glycemic index or load in women, stratified for parity. Fecundability represents the probability of conceiving within one month (28 days). Models were analysed using Cox proportional hazard models. Fecundability ratios were derived from the hazard ratios of the Cox proportional hazard models. **B)** Values represent the odds of subfertility (≥ 12 months to conceive) per SDS increase in the dietary glycemic index or load in women, stratified for parity. Models were analysed using logistic regression models.  Confounder model included: age, ethnicity, educational level, alcohol use, smoking, pre-pregnancy body mass index, and total energy intake.  *p-value < 0.05. | | | | | | |

**Supplementary Information S1.** Additional information on dietary intake data and processing

- 1. Additional information on processing of dietary intake data

For majority of the questions in the food frequency questionnaire (FFQ) participants were allowed to provide only one answer. When participants accidentally marked the wrong answer, they could correct their response by marking the correct answer box and fully coloring the answer box with the incorrect answer. During scanning of the questionnaires, when two answers were marked for a single question, the scanning systematically entered an “error” code. In the generated datasets, all instances of “error” codes were reviewed and cross-checked with the corresponding scanned questionnaires. When the system incorrectly recorded an “error” code, meaning the participant had fully colored one answer box and marked the correct answer box , the “error” code was recoded to the participants correct answer. When the system correctly recorded an "error" code, and the participant provided two adjacent answers, such as "2 tablespoons of cereal" and "3 tablespoons of cereal," the "error" code was recoded to the highest value given by the participant (in this example “3 tablespoons of cereal”). This choice was made to avoid complications with nutrient intake calculations, as using intermediate values (e.g., working with fractions) could pose difficulties. The preference was for the higher value, as individuals using self-reporting dietary instruments are more likely to underreport than overreport their intake (1,2). When the system correctly recorded an “error” code, and the participant provided two non-adjacent answers, the answer was considered missing. Finally, when the system correctly recorded an "error" code, and the participant answered "these four weeks I have not consumed this product," alongside another response option like "1 day per week," the choice was made to select "these four weeks I have not consumed this product". This decision was based on the likelihood that the mother's eating habits temporarily changed due to pregnancy-related symptoms in the first trimester (e.g., nausea/decreased appetite). These "error" codes were recoded to “these four weeks I have not consumed”. This recoding method was applied similarly to men's questionnaires to synchronize the process. In cases of uncertainty, the associated questionnaires were reviewed and discussed by two colleagues for verification.

1. Schoeller, DA. How accurate is self-reported dietary energy intake? (1990). Nutr Rev. 48:10:373-9.
2. Burrows, TL, Ho YY, Rollo ME, Collins CE. Validity of dietary assessment methods when compared to the method of doubly labeled water: a systematic review in adults. (2019). Front Endocrinol (Lausanne). 10:850.

1.2 Matching food items of the food frequency questionnaire with glycemic index values


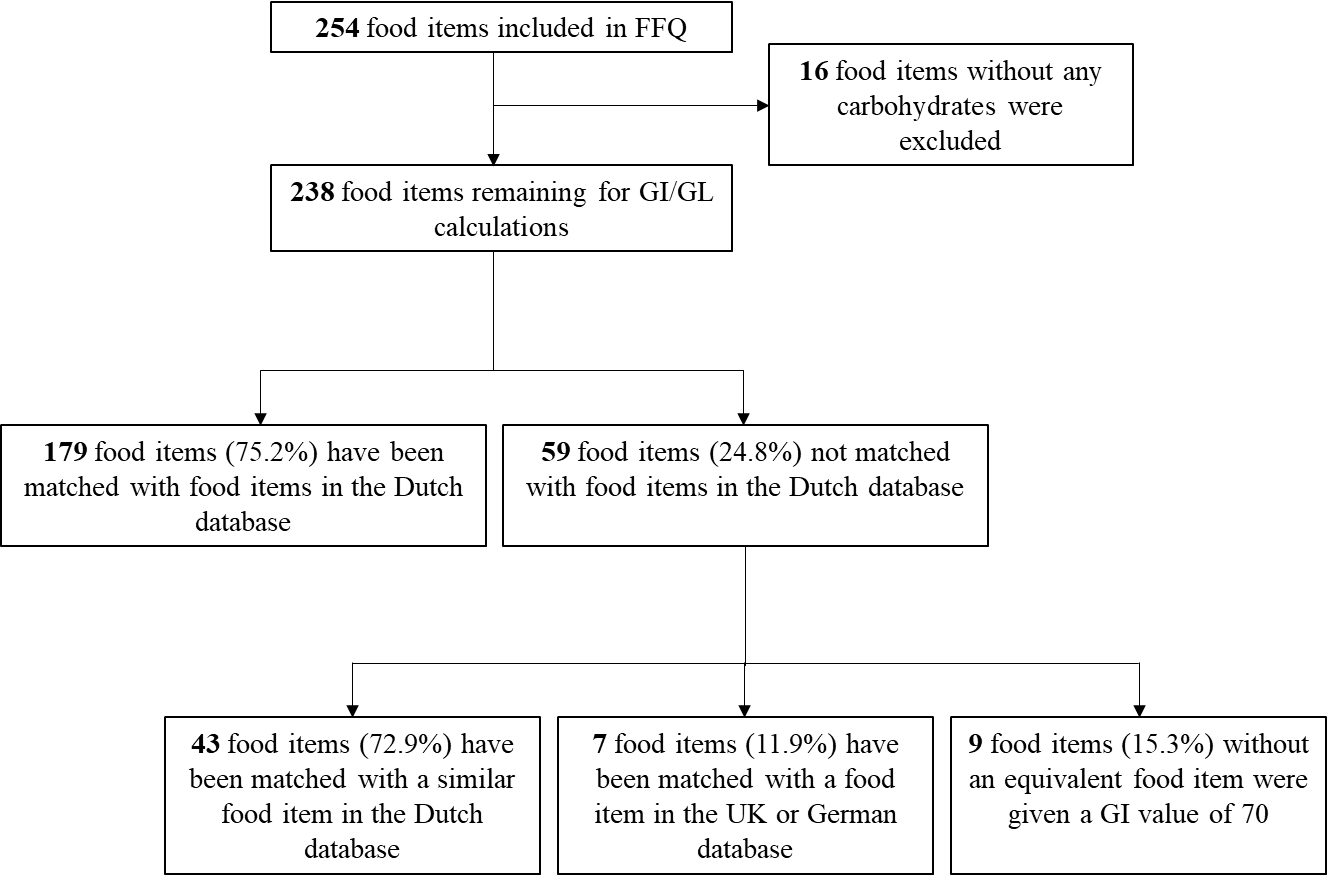


1.3 Dietary reference values for Dutch adults

|  | Women | | | Men | | |
| --- | --- | --- | --- | --- | --- | --- |
|  | **EAR** | **RDA** | **AI** | **EAR** | **RDA** | **AI** |
| ***Nutrient*** |  |  |  |  |  |  |
| Fibre (g/MJ) |  |  | 3.4 |  |  | 3.4 |
| Protein (g/kg/day) | 0.66 | 0.83 |  | 0.66 | 0.83 |  |
| Fats (en%) |  |  | 20-40 |  |  | 20-40 |
| Retinol Activity Equivalent (μg/day) | 525 | 680 |  | 615 | 800 |  |
| Vitamin B6 (mg/day) | 1.1 | 1.5 |  | 1.1 | 1.5 |  |
| Dietary Folate Equivalents (μg/day) | 200 | 300 |  | 200 | 300 |  |
| Vitamin B12 (μg/day) | 2.0 | 2.8 |  | 2.0 | 2.8 |  |
| Vitamin C (mg/day) | 50 | 75 |  | 60 | 75 |  |
| Vitamin D (μg/day) |  |  | 10 |  |  | 10 |
| Calcium (mg/day) | 750 | 950 |  | 750 | 950 |  |
| Iron (mg/day) | 7 | 16 |  | 6 | 11 |  |
| Magnesium (mg/day) |  |  | 300 |  |  | 350 |
| Zinc (mg/day) | 5.7 | 7 |  | 6.4 | 9 |  |
| EPA + DHA (g/day) |  |  | 0.2 |  |  | 0.2 |
| Dietary reference values established by the Dutch Health Council for adults. EAR: Estimated average requirement, RDA: Recommended daily allowance, AI: Adequate Intake, EPA: eicosapentaenoic acid, DHA: docosahexaenoic acid. | | | | | | |
